# Possible influence of mammographic density on local and locoregional recurrence of breast cancer

**DOI:** 10.1186/bcr3450

**Published:** 2013-07-11

**Authors:** Louise Eriksson, Kamila Czene, Lena Rosenberg, Keith Humphreys, Per Hall

**Affiliations:** 1 Department of Medical Epidemiology and Biostatistics, Karolinska Institutet, Nobels Väg 12A, S-171 77 Stockholm, Sweden; 2 Department of Radiology, Danderyds Hospital, Mörbygårdsvägen, S-182 88 Stockholm, Sweden

## Abstract

**Introduction:**

It is debated whether mammographic density gives rise to more aggressive cancers. We therefore aimed to study the influence of mammographic density on prognosis.

**Methods:**

This is a case-only study within a population-based case-control study. Cases were all postmenopausal women in Sweden with incident breast cancer, diagnosed 1993-1995, and aged 50-74 years. Women with pre-diagnostic/diagnostic mammograms were included (*n *= 1774). Mammographic density of the unaffected breast was assessed using a computer-assisted thresholding technique. The Cox proportional hazards model was used to study recurrence and survival with and without stratification on surgical procedure (breast-conserving surgery vs. mastectomy).

**Results:**

Percentage density (PD) was associated with both local and locoregional recurrence even after adjustment for established prognosticators; hazards ratio (HR) 1.92, p = 0.039, for local recurrence and HR 1.67, p = 0.033, for locoregional recurrence for women with PD≥25% compared to PD<25%. Stratification on surgical procedure showed that the associations were also present in mastectomized women. PD was neither associated with distant recurrence nor survival.

**Conclusions:**

High mammographic density is an independent risk factor of local and locoregional recurrence but is neither associated with distant metastasis nor survival. The relationships with local and locoregional recurrences were also present in women treated with mastectomy, indicating that they are not merely explained by density masking residual disease in women treated with breast-conserving surgery. Rather there appears to be a true association. Thus, mammographic density should possibly influence adjuvant therapy decisions in the future.

## Introduction

Mammographic density (MD) is one of the strongest risk factors for breast cancer. Women with highest density (>75%) have a fivefold increased risk compared with women with almost entirely fatty breasts (<5%) [1]. MD refers to the tissue composition of the breast; the epithelium and fibrous tissue are radiodense and appear white on a mammogram, whereas the fatty tissue is radiolucent and appears black. MD is often given as a percentage (percentage density, or PD), which is calculated by dividing the dense area by the total breast area. Consequently, larger amounts of fibroglandular tissue in relation to fatty tissue will lead to higher PD and vice versa.

Whether MD influences prognosis is not known. There are several possible mechanisms by which density could affect prognosis. MD has consistently been shown to be associated with the stromal composition of the breast [2], which is involved in tumor progression [3]. Hence, breast cancer originating in dense breasts may be associated with a more aggressive disease. Furthermore, MD decreases mammographic sensitivity since extensive density can hide tumors (a phenomenon referred to as masking). MD may thereby be associated with a poorer prognosis because of later detection. Masking may also influence the risk of recurrence, by hiding disease in the residual breast tissue of women who undergo breast-conserving surgery, thereby increasing the risk of recurrence. Lastly, MD may be associated with tumor characteristics or tumor subtype. Results are highly inconsistent, although most of these studies show no association [4-9]. Previous studies directly addressing the relationship between PD and survival show conflicting results [10-13], but the most recent and largest study [10] failed to detect an association between pre-diagnostic MD and survival. Hence, with this study, we wished to investigate whether MD influences prognosis, by studying its association with recurrence and survival.

## Materials and methods

### Study population

This study is an extension of a large case-control study among all Swedish residents who were born in Sweden and who were 50 to 74 years old at the time of enrollment (1 October 1993 to 31 March 1995). Details on data collection and subjects have been provided previously [14]. Women with incident primary invasive breast cancer were identified via the six Swedish Regional Cancer Registries (*n *= 3,979), and 84% (*n *= 3,345) participated. However, 19 cases were diagnosed outside of the study period, one case had a diagnosis other than breast cancer, and 58 cases had non-invasive breast cancer, rendering them ineligible and leaving 3,267 women in the study.

For this study, the inclusion criteria were further refined to include only post-menopausal women who had no prior diagnosis of cancer other than non-melanoma skin cancer (see Eriksson *et al*. [6] for details). The study base thus consisted of 2,720 breast cancer cases.

Details on mammography retrieval have been provided elsewhere [6]. For the eligible participants in this study, we managed to collect mammograms for 2,046 women (75%). However, 107 women who had only post-diagnostic mammograms were excluded, as were 65 women lacking mammograms of the breast contralateral to the tumor and 79 women lacking post-menopausal mammograms. The latter exclusion is due to the fact that MD may differ histologically in pre- and post-menopausal women [15] and also may be affected differentially by hormones [16]. Lastly, images of poor quality were omitted, excluding 21 women. The median difference from date of mammography to study entrance was 50 days.

Values of descriptive statistics (means and proportions) for women excluded because of the factors above did not differ significantly from those for included women (*n *= 1,774) for body mass index (BMI), breast cancer heredity, previous benign breast disease, age at menarche, oral contraceptive use, age at first birth, parity, breast feeding, age at menopause, and hormone replacement therapy (HRT) use. However, there was a difference in age at study entrance (62.9 years for included women and 63.6 years for excluded women; *P *= 0.015).

Approval for the study was given by the ethical review boards in the respective regions in which the subjects were based: the Regional Ethical Review Board in Gothenburg, the Regional Ethical Review Board in Linköping, the Regional Ethical Review Board in Malmö-Lund, the Regional Ethical Review Board in Stockholm (at Karolinska Institutet), the Regional Ethical Review Board in Umeå, and the Regional Ethical Review Board in Uppsala. Prior to participation via a mailed questionnaire, written consent was obtained from all patients.

### Data collection and classification

Data on sociodemographic, anthropometric, hormonal, and lifestyle factors were collected by means of a postal questionnaire. Because date of mammography was prior to study entrance, the variables of age, menopausal status, and HRT use were reassessed according to date of mammography. This was not possible for BMI as we had information on BMI at study entrance and 1 year prior to study entrance only. However, it has previously been shown that inter-individual variations in BMI are small [17], and the difference in BMI at study entrance and 1 year prior to this was 0.05 units (standard deviation of 1.2) for our study participants.

HRT use was classified as 'current', 'former', or 'never' to indicate how recent it was. Because the influence of HRT on MD is supposed to diminish within 3 weeks of cessation [18], former users were those who discontinued use of HRT more than 1 month prior to date of mammography. All compounds, modes of administration, and potencies - except for low-potency, estrogen-only pharmaceuticals, since these have not been shown to increase breast cancer risk - were included in the HRT variable [14].

We used the national registration numbers to retrieve patient records and register information. Between 2000 and 2002, we collected information on primary surgery, adjuvant treatment (endocrine therapy, chemotherapy, and radiotherapy), tumor characteristics, possible recurrence, and reason for diagnostic mammography from surgical and oncological patient records throughout Sweden.

We collected information on emigrations from the Swedish National Population Register and on the date and cause of death until 31 December 2008 from the Swedish Causes of Death Registry. The latter registry covers all residents in Sweden with essentially no missing deaths and has been shown to correctly classify 98% of breast cancer deaths [19]. The follow-up of vital status is thus virtually complete.

Grade was classified according to the Nottingham histologic grade or the Bloom-Richardson scale into three groups. Tumors were considered estrogen receptor (ER)-positive or progesterone receptor (PR)-positive if they contained at least 0.05 fmol receptor/μg DNA or at least 10 fmol receptor/mg protein.

Type of surgery was classified as breast-conserving surgery or mastectomy independent of axillary surgery because 98% of our study participants had axillary lymph node clearance carried out. Local recurrence included recurrence in the residual ipsilateral breast, scar tissue, or chest wall. Locoregional recurrence included both local recurrences and recurrences in regional lymph nodes. During the collection of follow-up information from clinical records, the study subjects' follow-up period was marked as complete or incomplete depending on whether follow-up information was complete or missing until date of retrieval. The follow-up time for recurrence variables was thus computed as follows: If the specific recurrence occurred, the follow-up time was computed from the date of diagnosis to the date of that specific recurrence; if the specific recurrence did not occur and follow-up information from medical records was considered complete, the follow-up time was computed from the date of diagnosis to the date of medical record retrieval or the date of death, whichever came first; if information on follow-up was considered incomplete, the follow-up time was computed from the date of diagnosis to the last documented date in the medical record or the date of death, whichever came first.

### Mammographic density data

Film mammograms of the medio-lateral oblique view were digitized by using an Array 2905HD Laser Film Digitizer (Array Corporation, Tokyo, Japan), which covers a range of 0 to 4.7 optical density. The medio-lateral oblique view was used since this was the routine view used at mammography screening in Sweden. The density resolution was set at 12-bit spatial resolution. We used Cumulus, a computer-assisted thresholding technique, to assess density [20] of the mammogram contralateral to the tumor. For each image, a trained observer (LE) set the appropriate gray-scale threshold levels that defined the edge of the breast and that distinguished dense from non-dense tissue. The software calculated the total number of pixels within the entire region of interest and within the region identified as dense. The PD was then calculated from these values (dense area divided by total breast area). The images were measured together with approximately the same number of images for healthy women, and the reader was blinded to case-control status and, naturally, to prognosis. A random 10% of the images were included as replicates to assess the intra-observer reliability, which was high (R^2 ^of 0.92).

### Statistical analysis

As in the report by Eriksson and colleagues [6], density was used as a binary variable throughout because it is more clinically relevant than a continuous measure and more easily interpreted. The cutoff was set at 25% defining the highest quartile in our cohort, i.e. 25% of the study population had a PD of at least 25%. We included post-menopausal women only. The MD in these women is lower and more homogeneous than in a combined pre- and post-menopausal population. We did not categorize PD according to the commonly used categories introduced by Boyd and colleagues [21], since the numbers of women in the highest categories were relatively few. Both recurrences and deaths are relatively rare events, and we wanted a fairly large number of women in each density category in order to avoid committing a type II error due to low power.

The descriptive characteristics of cases with high density versus cases with lower density were compared by using chi-squared tests for categorical variables and Student *t *tests for continuous variables. We also carried out age-adjusted analyses pertaining to the above by using logistic regression.

The survival analyses were carried out by using the Cox proportional hazards model. We made use of two models. The first model was carried out to attempt to study the association between MD and recurrence and survival variables. We therefore included only age (continuous), BMI (continuous), and HRT use (categorical) in this model since these variables are strongly associated with MD and breast cancer risk [22] and may influence prognosis [23-26]. The second model was carried out to show whether any possible associations were independent of established prognosticators. Therefore, in addition to including the variables in the first model, the second model included the following variables: tumor size (continuous), lymph node metastasis (continuous), ER status (categorical), PR status (categorical), grade (categorical), and mode of detection (binary: screening-detected versus non-screening-detected cancer) [27,28]. Whereas less than 5% of our study population were missing information on mode of detection, tumor size, and presence of lymph node metastasis, respectively, approximately 30% were missing information on grade and ER and PR status. We thus added a missing category to these variables. All analyses were carried out by using the statistical software, STATA 12.1 (StataCorp LP, College Station, TX, USA).

## Results

The mean PD was 18%. Age at mammography (*P *<0.0001), BMI (*P *<0.0001), previous oral contraceptive use (*P *<0.001), parity and age at first birth (*P *= 0.005), HRT use (*P *<0.001), and previous benign breast disease (*P *<0.001) were all associated with PD (Table 1). After adjustment for age, PD was still statistically significantly associated with BMI (*P *<0.001), parity and age at first birth (*P *= 0.001), HRT use (*P *<0.001), and previous benign breast disease (*P *<0.001) as well as age at menopause (*P *= 0.046).

**Table 1:** Descriptive statistics based on percentage density

	All cases (*n *= 1,774)			
	
	Unadjusted			Age-adjusted
	
	PD <25% (*n *= 1,329)	PD ≥25% (*n *= 445)	*P *value	*P *value
Age at mammography, years	63.1	59.0	<0.001	

Body mass index	26.4	23.9	<0.001	<0.001

Age at menarche, years	13.6	13.5	0.719	0.310

Oral contraceptive use			<0.001	0.484

Yes	410 (31%)	184 (41%)		

No	914 (69%)	261 (59%)		

Parity and age at first birth			0.005	0.001

Nulliparous	173 (13%)	79 (18%)		

Parity ≤2 and age at first birth ≤25	386 (29%)	136 (31%)		

Parity ≤2 and age at first birth >25	403 (30%)	144 (32%)		

Parity >2 and age at first birth ≤25	272 (20%)	63 (14%)		

Parity >2 and age at first birth >25	96 (7%)	23 (5%)		

Breast feeding			0.174	0.069

Yes	940 (80%)	316 (77%)		

No	229 (20%)	93 (23%)		

Age at menopause, years	50.4	50.5	0.506	0.046

HRT use			<0.001	<0.001

Never	974 (74%)	244 (56%)		

Past	70 (5%)	70 (16%)		

Current	268 (20%)	124 (28%)		

Previous benign breast disease			<0.001	<0.001

Yes	161 (12%)	89 (20%)		

No	1,163 (88%)	354 (80%)		

Breast cancer heredity			0.633	0.829

Yes	200 (15%)	64 (15%)		

No	1,091 (85%)	376 (85%)		

Values other than *P *values are presented as number (percentage) or mean. HRT, hormone replacement therapy; PD, percentage density.

There were statistically significant differences between women with a PD of at least 25% compared with women with a PD of less than 25% for mode of detection (*P *= 0.011) and tumor size (*P *= 0.007) as well as a tendency for women with dense breasts to develop locoregional recurrence more often (*P *= 0.056) (Table 2).

**Table 2:** Description of mode of detection, tumor characteristics, treatment, and prognosis variables based on percentage density

	All cases (*n *= 1,774)	PD <25% (*n *= 1,329)	PD ≥25% (*n *= 445)	*P *value
Mode of detection				0.011

Screening-detected	1,115 (64%)	867 (67%)	248 (57%)	

Referral for check-up, no symptoms	25 (1%)	18 (1%)	7 (2%)	

Referral due to HRT use, no symptoms	21 (1%)	15 (1%)	6 (1%)	

Symptomatic	551 (32%)	384 (30%)	167 (39%)	

Referral, other reason	18 (1%)	13 (1%)	5 (1%)	

Tumor size, mm	16.8	16.4	18.0	0.007

Lymph node metastasis				0.581

Positive	480 (28%)	362 (29%)	118 (27%)	

Negative	1,221 (72%)	905 (71%)	316 (73%)	

Histopathologic classification				0.230

Ductal	1,268 (73%)	957 (73%)	311 (72%)	

Lobular	199 (11%)	140 (11%)	59 (14%)	

Other	268 (15%)	206 (16%)	62 (14%)	

ER status				0.733

ER-positive	971 (79%)	727 (79%)	244 (78%)	

ER-negative	256 (21%)	189 (21%)	67 (22%)	

PR status				0.779

PR-positive	808 (67%)	602 (67%)	206 (68%)	

PR-negative	396 (33%)	298 (33%)	98 (32%)	

Grade				0.382

1	197 (17%)	151 (17%)	46 (15%)	

2	510 (43%)	382 (44%)	128 (42%)	

3	475 (41%)	342 (39%)	133 (43%)	

Chemo- and endocrine therapy				0.507

Chemotherapy only	51 (3%)	34 (3%)	17 (4%)	

Endocrine therapy only	788 (46%)	590 (45%)	198 (46%)	

Both	81 (5%)	59 (5%)	22 (5%)	

None	812 (47%)	615 (47%)	197 (45%)	

Locoregional radiotherapy				0.425

Yes	1,098 (63%)	816 (63%)	282 (65%)	

No	633 (37%)	481 (37%)	152 (35%)	

Type of breast operation				0.580

Breast-conserving surgery	1,046 (61%)	786 (61%)	260 (60%)	

Mastectomy	683 (40%)	509 (39%)	174 (40%)	

No surgery	3 (0%)	3 (0%)	0 (0%)	

Local recurrence				0.304

Yes	62 (4%)	43 (3%)	19 (4%)	

No	1,668 (96%)	1,253 (97%)	415 (96%)	

Locoregional recurrence				0.056

Yes	103 (6%)	69 (5%)	34 (8%)	

No	1,627 (94%)	1,227 (95%)	400 (92%)	

Distant metastasis				0.361

Yes	187 (11%)	135 (10%)	52 (12%)	

No	1,544 (89%)	1,162 (90%)	382 (88%)	

Breast cancer death within 5 years				0.405

Yes	124 (7%)	89 (7%)	35 (8%)	

No	1,649 (93%)	1,239 (93%)	410 (92%)	

Breast cancer death within 10 years				0.204

Yes	213 (12%)	152 (11%)	61 (14%)	

No	1,560 (88%)	1,176 (89%)	384 (86%)	

Death within 5 years independent of cause				0.902

Yes	174 (10%)	131 (10%)	43 (10%)	

No	1,599 (90%)	1,197 (90%)	402 (90%)	

Death within 10 years independent of cause				0.599

Yes	358 (20%)	272 (20%)	86 (19%)	

No	1,415 (80%)	1,056 (80%)	359 (81%)	

Values are presented as number (percentage) or mean. ER, estrogen receptor; HRT, hormone replacement therapy; PD, percentage density; PR, progesterone receptor.

Results from survival analyses are reported in Table 3. PD was associated with local and locoregional recurrence both before (hazard ratio (HR) for local recurrence 1.99, 95% confidence interval (CI) 1.09 to 3.66; HR for locoregional recurrence 1.84, 95% CI 1.16 to 2.91) and after adjustment for established prognosticators (HR for local recurrence 1.92, 95% CI 1.03 to 3.57; HR for locoregional recurrence 1.67, 95% CI 1.04 to 2.69). No associations between PD, distant metastasis, breast cancer-specific survival, and overall survival were observed. After finding associations between PD and local and locoregional recurrence, we investigated whether there was a dose-response relationship by categorizing PD into quartiles. We found support of such a relationship (Table 4).

**Table 3:** Association of percentage density with breast cancer recurrence and survival

	Model 1^a^			Model 2^b^		
	
	HR^c^	95% CI	*P *value	HR^c^	95% CI	*P *value
Local recurrence 62 events Total time at risk = 9,683 years	1.99	1.09-3.66	0.026	1.92	1.03-3.57	0.039

Locoregional recurrence 103 events Total time at risk = 9,606 years	1.84	1.16-2.91	0.010	1.67	1.04-2.69	0.033

Distant recurrence 181 events Total time at risk = 9,618 years	1.28	0.90-1.82	0.170	1.08	0.74-1.56	0.698

5-year breast cancer-specific survival 124 events Total time at risk = 8,489 years	1.25	0.82-1.92	0.299	0.97	0.61-1.54	0.908

10-year breast cancer-specific survival 213 events Total time at risk = 16,016 years	1.34	0.97-1.85	0.080	1.08	0.77-1.53	0.644

Overall 5-year survival 174 events Total time at risk = 8,489 years	1.07	0.73-1.55	0.742	0.89	0.60-1.32	0.554

Overall 10-year survival 358 events Total time at risk = 16,016 years	1.14	0.87-1.48	0.343	0.99	0.75-1.29	0.921

^a^Adjusted for age, body mass index (BMI), and hormone replacement therapy (HRT) use;

^b^adjusted for age, BMI, HRT use, mode of detection, tumor size, lymph node metastasis, estrogen receptor status, progesterone receptor status, and grade;

^c^hazard ratio (HR) comparing percentage density (PD) ≥25% to PD <25%. CI, confidence interval.

**Table 4:** Association between mammographic density categorized into quartiles and local and locoregional recurrence

	Local recurrence			Locoregional recurrence		
	
	HR	95% CI	*P *value	HR	95% CI	*P *value
Model 1^a^			0.008			0.012

PD, 1st quartile^b^	1.00 (Ref.)			1.00 (Ref.)		

PD, 2nd quartile^c^	1.72	0.78-3.78		1.43	0.79-2.60	

PD, 3rd quartile^d^	1.91	0.86-4.24		1.34	0.72-2.50	

PD, 4th quartile^e^	3.14	1.38-7.16		2.36	1.27-4.39	

Model 2^f^			0.017			0.035

PD, 1st quartile^b^	1.00 (Ref.)			1.00 (Ref.)		

PD, 2nd quartile^c^	1.80	0.80-4.05		1.38	0.75-2.53	

PD, 3rd quartile^d^	1.78	0.77-4.11		1.29	0.68-2.45	

PD, 4th quartile^e^	3.03	1.28-7.17		2.10	1.11-3.97	

^a^Adjusted for age, body mass index (BMI), and hormone replacement therapy (HRT) use;

^b^percentage density (PD) <6.46, *n *= 443;

^c^6.46 ≤ PD <13.66, *n *= 444;

^d^PD 13.66 ≤ PD <25.07, *n *= 444;

^e^PD ≥25.07, *n *= 443;

^f^adjusted for age, BMI, HRT use, mode of detection, tumor size, lymph node metastasis, estrogen receptor status, progesterone receptor status, and grade. CI, confidence interval; HR, hazard ratio; Ref., reference value.

The associations described in Tables 3 and 4 between PD and local and locoregional recurrence could be due to the masking of residual disease by MD in women who underwent breast-conserving surgery. We therefore redid the analyses pertaining to PD, local, and locoregional recurrence, stratifying on surgical procedure (Table 5). For women who underwent mastectomy, the HRs for local and locoregional recurrence were 2.93 (95% CI 1.03 to 8.39) and 2.16 (95% CI 1.02 to 4.54), respectively, after full adjustment. For women who underwent breast-conserving surgery, the HRs for local and locoregional recurrence were 1.48 (95% CI 0.67 to 3.25) and 1.57 (95% CI 0.83 to 2.97), respectively, after full adjustment.

**Table 5:** Association between percentage density (≥25% compared with <25%) and local recurrence stratified on surgical method

	Breast-conserving surgery			Mastectomy		
Local recurrence	41 events Time at risk = 5,959 years			21 events Time at risk = 3,734 years		

	HR	CI	*P *value	HR	CI	*P *value

Model 1^a^	1.53	0.70-3.31	0.286	3.44	1.26-9.38	0.016

Model 2^b^	1.48	0.67-3.25	0.334	2.93	1.03-8.39	0.045

Locoregional recurrence	61 events Time at risk = 5,924 years			42 events Time at risk = 3,690 years		

	HR	CI	*P *value	HR	CI	*P *value

Model 1^a^	1.45	0.78-2.70	0.242	2.69	1.34-5.43	0.006

Model 2^b^	1.57	0.83-2.97	0.166	2.16	1.02-4.54	0.044

^a^Adjusted for age, body mass index (BMI), and hormone replacement therapy (HRT) use.

^b^Adjusted for age, BMI, HRT use, mode of detection, tumor size, lymph node metastasis, estrogen receptor status, progesterone receptor status, and grade. CI, confidence interval; HR, hazard ratio.

Although we used PD as a binary variable, we also carried out analyses by using PD as a continuous measure. This did not change the interpretation of the results; for example, *P *= 0.008 for the association between continuous PD and locoregional recurrence before adjustment for established prognosticators and *P *= 0.032 after full adjustment.

## Discussion

MD is one of the strongest risk factors for breast cancer, and we have shown that it also increases risk of local and locoregional recurrence after diagnosis of invasive breast cancer. We found no association between density and distant metastasis or between density and survival in our post-menopausal study population.

There was support of a dose-response relationship between PD and both local and locoregional recurrence. Women in the highest PD quartile (PD ≥25.07) had a threefold higher risk of local recurrence and twofold higher risk of locoregional recurrence compared with women in the lowest PD quartile (PD <6.47; Table 4), independent of established breast cancer prognosticators. The association between PD and locoregional recurrence is in agreement with the two previously published studies investigating this relationship in women with invasive breast cancer [13,29], although they both [13,29] were limited to women who had undergone breast-conserving surgery. Even though most locoregional recurrences occur in women who have undergone breast-conserving surgery, there remains a risk of developing recurrence in the scar tissue, chest wall, and regional lymph nodes after mastectomy. Furthermore, if an association between MD and locoregional recurrence is restricted to women who underwent breast-conserving surgery, then it could simply be because density masked residual disease in the conserved breast. This reservation is especially important in our study since we lacked information on the status of the surgical margin. We thus stratified on type of breast surgery and found that the relationship also was present in the group of women who underwent mastectomy, undermining this hypothesis. We do not wish to draw any conclusions on whether type of surgery influences the risk of recurrence, since differences in personal and clinical characteristics (for example, age, tumor size, and comorbidity) will be different in the groups selected for the two different surgical techniques. This will lead to confounding by indication.

We did not see an association between density and distant recurrence or with survival. Distant metastasis is closely linked with breast cancer mortality, whereas locoregional recurrence has a moderate impact on survival [30]. Since we had only a 10-year follow-up of survival, we cannot exclude the possibility that there is a relationship between PD and long-term survival or that there is a small effect of PD on survival, requiring even larger studies to identify the association. However, the lack of an association between MD, distant metastasis, and survival is in accordance with both previous studies pertaining to MD and distant metastasis [13,29] and two of three studies on MD and survival [10,11,13]. The conflicting study relating to density and prognosis [12] showed a lower case fatality for women with high density compared with women with low density. However, because only age was adjusted for, this relationship is most likely due to confounding.

The mean density was relatively low in this study population since all women were post-menopausal when the mammograms were taken. The mean age at mammography was thus relatively high (approximately 62 years of age). The study population was originally population-based. Hence, we had information on breast cancer risk factors on 84% of all women who were 50 to 74 years of age and whose invasive breast cancer was diagnosed in Sweden during 1 October 1993 to 31 March 1995, and we were able to compare this information with the women who were not included in the present study. We found that there were no differences in risk factors (for example, BMI, family history, parity, breast feeding, and HRT use) except for a difference in age at study entrance of the original study (62.9 years for included women compared with 63.6 years for excluded women; *P *= 0.015). Thus, our density measurements should be representative for a post-menopausal cohort. Because the reader was blinded to outcomes, any measurement error would be a non-differential misclassification of exposure and thus would only attenuate results. However, the reader who was trained by Norman Boyd, an expert on MD and one of the developers of Cumulus [20], regularly calibrated herself against his measurements and had a high intra-observer correlation (R^2 ^= 0.92), which should minimize exposure misclassification.

Our study has several strengths: the population-based design, size, and detailed covariate information, including mode of detection, quantitative semi-automated density measurements to minimize exposure misclassification, and long and virtually complete follow-up of vital status and cause of death. A limitation of our study was that the study population was restricted to post-menopausal women; hence, our results may not be applicable to pre-menopausal populations. We also lacked information on HER2 status and Ki67 since they were not clinically in use in Sweden at the time breast cancer was diagnosed in our participants. Both HER2-status and Ki67 influence prognosis [31,32]. However, for these factors to have influenced our results, they must be associated with MD, and at present there is no evidence of such an association [7,9,33,34].

How could MD influence risk of local and locoregional recurrence but not distant metastasis? We can only speculate on the mechanisms behind this. MD could increase the risk of self-seeding, a process in which disseminated tumor cells return and colonize the primary tumor site [35]. In contrast to metastasis, which requires the ability to enter, survive, and colonize a new site, self-seeding demands little or no adaptation since the circulating tumor cell returns to a familiar environment [36]. Since disseminated tumor cells can reside in a dormant manner in new organs without giving rise to metastatic colonies, seeding of the primary site does not require manifest metastasis [36]. This has been presented as an explanation for why local recurrence can follow a complete eradication of the primary tumor [35].

MD has been hypothesized to give rise to more aggressive tumors due to the increased stromal composition of the breast and increased deposition of collagen [15,37-39]. Despite this, we and others [13,29] were not able to show an association between MD and risk of distant metastasis. Perhaps, however, density does increase risk of dissemination, but the organ of preference is the primary tumor site.

## Conclusions

High MD increases the risk of local and locoregional recurrence but not distant recurrence or survival. The relationships with local and locoregional recurrences were even present in women who underwent mastectomy and therefore are not due merely to the masking of residual disease by MD in women who underwent breast-conserving surgery. Instead, there appears to be a true association. MD seems to be an independent risk factor of local and locoregional recurrence and should possibly influence adjuvant therapy decisions in the future.

## Abbreviations

BMI: body mass index; CI: confidence interval; ER: estrogen receptor; HR: hazard ratio; HRT: hormone replacement therapy; MD: mammographic density; PD: percentage density; PR: progesterone receptor.

## Competing interests

The authors declare that they have no competing interests.

## Authors' contributions

LE participated in the collection of the data, carried out the density measurements, carried out the statistical analyses with help from KH, and wrote the first draft of the manuscript. LR participated in the collection of the data. All authors (KC, LE, PH, KH, and LR) participated in the study design, interpretation of results, and the revision of the manuscript and read and approved the final manuscript.
